# The effect of membrane thickness on the membrane permeabilizing activity of the cyclic lipopeptide tolaasin II

**DOI:** 10.3389/fmolb.2022.1064742

**Published:** 2022-12-23

**Authors:** Jessica Steigenberger, Catherine Mergen, Vic De Roo, Niels Geudens, José C. Martins, Heiko Heerklotz

**Affiliations:** ^1^ Department of Pharmaceutics, Institute of Pharmaceutical Sciences, University of Freiburg, Freiburg, Germany; ^2^ NMR and Structure Analysis Research Group, Department of Organic and Macromolecular Chemistry, Ghent University, Ghent, Belgium; ^3^ Leslie Dan Faculty of Pharmacy, University of Toronto, Toronto, ON, Canada; ^4^ Signaling Research Centers BIOSS and CIBSS, University of Freiburg, Freiburg, Germany

**Keywords:** cyclic lipopeptides, tolaasin II, equi-activity analysis, membrane thickness, packing mismatch, membrane stability

## Abstract

Tolaasin II is an amphiphilic, membrane-active, cyclic lipopeptide produced by *Pseudomonas tolaasii* and is responsible for brown blotch disease in mushroom. To better understand the mode of action and membrane selectivity of tolaasin II and related lipopeptides, its permeabilizing effect on liposomes of different membrane thickness was characterized. An equi-activity analysis served to distinguish between the effects of membrane partitioning and the intrinsic activity of the membrane-bound peptide. It was found that thicker membranes require higher local peptide concentrations to become leaky. More specifically, the mole ratio of membrane-bound peptide per lipid needed to induce 50% leakage of calcein within 1 h, R_e_^50^, increased monotonically with membrane thickness from 0.0016 for the 14:1 to 0.0070 for the 20:1 lipid-chains. Moreover, fast but limited leakage kinetics in the low-lipid regime were observed implying a mode of action based on membrane asymmetry stress in this time and concentration window. While the assembly of the peptide to oligomeric pores of defined length along the bilayer *z*-axis can in principle explain inhibition by increasing membrane thickness, it cannot account for the observed limited leakage. Therefore, reduced intrinsic membrane-permeabilizing activity with increasing membrane thickness is attributed here to the increased mechanical strength and order of thicker membranes.

## Introduction

Cyclic lipopeptides (CLiPs) can be considered a special class of antimicrobial peptides (AMPs), which are produced by a wide variety of organisms for host defense and other functions. Their common feature is their amphiphilic character, enabling them to bind, alter, and potentially permeabilize the lipid matrix of target membranes. CLiPs, as discussed here, are produced by bacteria, including *Bacillus* and *Pseudomonas* species (Raaijmakers et al., 2010; Schneider et al., 2014; Balleza et al., 2019). Their structure combines specific features such as *d* -amino acids and other non-proteinogenic amino acids, cyclization, and lipidation (Geudens and Martins, 2018; Götze and Stallforth, 2020), which are now considered as optimization parameters for AMPs to improve their stability and their activity (Huan et al., 2020).

The great versatility of CLiP activities is medically and technically interesting in several respects (Schneider et al., 2014; Patel et al., 2015). Their medical use as another class of antibiotics has been receiving much attention. In addition to established drugs such as daptomycin or polymyxin, new CLiP leads are continuously being identified and developed to fight antibiotic resistance (Marner et al., 2020; Roberts et al., 2022; Wang et al., 2022). Moreover, CLiPs inhibiting, for example, pathogenic fungi are used for crop protection (Crouzet et al., 2020; Malviya et al., 2020; Blake et al., 2021). In contrast, the CLiP studied here, tolaasin II, is involved in the pathogenic effects of its producer, *Pseudomonas tolaasii*, against mushroom cultures (Soler-Rivas et al., 1999), strawberries, cauliflower, and tobacco. Its activity to permeabilize lipid membranes (Rainey et al., 1991; Coraiola et al., 2006) contributes to the induction of brown blotch disease of fungi. The optimal handling of these beneficial or detrimental effects of CLiPs profits from a better understanding of the mechanisms governing their membrane-perturbing activity and selectivity for certain target membranes. In this context, it is important to recall that AMPs (including CLiPs) have been described to act by a plethora of potential mechanisms and combinations thereof (Huang, 2006; Nguyen et al., 2011; Wimley and Hristova, 2019).

Our primary aim is to better understand the molecular properties of a CLiP and the target membrane that control selective action. After addressing the effect of the length of the fatty acid chain (Steigenberger et al., 2021) and the charge of the CLiPs (Steigenberger et al., 2022), it was tested here how the thickness of the target membrane affects activity. Tolaasin II was chosen as an example because it is a typical representative of a large structural group of CLiPs in which a lipid chain, an amphiphilic helix, and a small and positively charged closed loop form a “golf-club\SPACE motif” (Jourdan et al., 2003), and also because of the great economic importance of the peptide as a virulence factor for e.g. brown blotch disease (Soler-Rivas et al., 1999). To probe membrane thickness effects, an established strategy was followed and the activity of tolaasin II against membranes of a homologous series of phosphatidylcholines with symmetric, monounsaturated chains with lengths from 14:1 to 20:1 was tested (Ashrafuzzaman et al., 2008; Bobone et al., 2012; Bobone et al., 2013; Lee et al., 2018; Henderson et al., 2019; Kim et al., 2021). These lipids form stable, fluid bilayers at ambient temperature, are well characterized (Kucerka et al., 2008; Kucerka et al., 2009; Frallicciardi et al., 2022) and commercially available at high purity. In this way, Stella and coworkers (Bobone et al., 2013) demonstrated that alamethicin and trichogen GA IV (AMPs most presumably acting by forming specific, oligomeric “barrel-stave” pores) became continuously less active with increasing target membrane thickness by varying phospholipid chains from 14:1 to 20:1. The gradual loss of activity with increasing membrane thickness was much more pronounced for the shorter trichogen GA IV molecule. This was explained by the increasing energy requirement of membrane deformations to hydrophobically match peptide pores shorter than the relaxed membrane thickness (Bobone et al., 2013). A similar effect of membrane thickness was also observed for pHD15 (Kim et al., 2021) and maculatin 1.1 (Lee et al., 2018), other AMPs that are proposed to span the membrane to induce leakage.

However, gradual inhibition of AMP-induced leakage with increasing membrane thickness is not sufficient to assume a barrel-stave mechanism, as demonstrated by mastoparan-X, an AMP assumed to act *via* local monolayer curvature stress and subsequent toroidal pore formation (Bobone et al., 2012). Although such a lipid-lined pore is not limited by the length of the peptide, it has been shown that increasing membrane thickness counteracts pore formation by mastoparan-X as well. Inhibition of the membrane-activity of AMPs such as protegrin1 (Henderson et al., 2019) and the CLiPs surfactin (Carrillo et al., 2003), fengycin (Fiedler and Heerklotz, 2015), tolaasin and sessilin (Ferrarini et al., 2022) by cholesterol has also been explained by cholesterol-induced increase in membrane thickness, order and mechanical stability. In contrast, gramicidin S, a cyclic AMP also acting by unspecific membrane defects, showed no systematic change in activity with lipid chain lengths ranging from 18:1 through 22:1, indicating that “inhibition by increased thickness” is not a general trend (Ashrafuzzaman et al., 2008).

A third principal mode of action of AMPs is based on asymmetry stress between the outer membrane leaflet, which is expanded by inserting peptide, and the inner one preferring to maintain its area (Heerklotz, 2001). Its hallmark in leakage experiments using liposomes as model membranes is the occurrence of transient, limited leakage that releases part of an entrapped dye within a few minutes or less (Pokorny and Almeida, 2004; Wimley, 2015). Leakage through a lipid-lined membrane defect inevitably also allows for the transmembrane flip of some lipids, which reduces the leakage-inducing lipid asymmetry. As the stress is relaxed below a threshold for membrane failure, the liposome membranes anneal and the remaining enclosed dye remains trapped. Although the CLiP studied here, tolaasin II, is able to form oligomeric structures that may allow for leakage (Coraiola et al., 2006; Lo Cantore et al., 2006; Andolfi et al., 2008), the asymmetry-based mode of action is found to dominate membrane permeabilization in the experiments presented here.

The key question addressed in this study is how asymmetry-based leakage depends on membrane thickness. Both scenarios, an increase or decrease, appear realistic. On the one hand, a recent study showed that the intrinsic activity of pseudodesmin A (another *Pseudomonas* CLiP) decreases with increasing acyl chain length of the CLiP at constant membrane thickness. This could be related to the curvature stress (Gruner, 1985; Israelachvili, 2011) that builds up when the hydrophobic volume of CLiP is insufficient to fill the space created by its interfacial requirement and the hydrophobic thickness of the lipid layer (Heerklotz, 2008). If this mismatch dominates the interaction of tolaasin II with the lipids tested, its intrinsic activity should be expected to increase with membrane thickness. If, on the other hand, the overall stability and order of the membrane were decisive, the intrinsic activity of tolaasin II would have to decrease by increasing the membrane thickness, as in the above examples.

## Materials and methods

### Materials

The phospholipids 1,2-dimyristoleoyl-sn-glycero-3-phosphocholine (14:1 PC), 1,2-dipalmitoleoyl-sn-glycero-3-phosphocholine (16:1 PC), 1,2-dioleoyl-sn-glycero-3-phosphocholine (18:1 PC), and 1,2-dieicosenoyl-sn-glycero-3-phosphocholine (20:1 PC) were purchased from Avanti Polar Lipids (Alabaster, AL, United States) dissolved in chloroform (10 mg/ml). The phospholipid 1-palmitoyl-2-oleoyl-*sn*-glycero-3-phosphocholine (POPC) as dry powder was a gift from Lipoid GmbH (Ludwigshafen, Germany).

Ammonium molybdate tetra-hydrate, Fiske-Subbarow reducer, H_2_O_2_ 30% wt, and H_2_SO_4_ (>98%), purchased from Sigma-Aldrich (St. Louis, MO, United States) and K_2_HPO_4_, bought from VWR (Leuven, Belgium) were used for lipid quantification.

NaCl, NaOH, HCl, and Tris (hydroxymethyl)aminomethane (Tris) from Carl Roth GmbH (Karlsruhe, Germany) and ethylenediaminetetraacetic acid (EDTA) from Sigma-Aldrich (St. Louis, MO, United States) were used for the preparation of the Tris buffer.

The solvents chloroform and dimethyl sulfoxide (DMSO) were used for lipid film and further sample preparation and bought from Carl Roth GmbH (Karlsruhe, Germany).

Calcein and Ludox®-HS 40 colloidal silica used for fluorescence analysis were purchased from Sigma-Aldrich (St. Louis, MO, United States) and ultrapure water used for the preparation of all solutions was prepared by the arium^®^ pro system (Sartorius AG, Göttingen, Germany).

**FIGURE 1:**
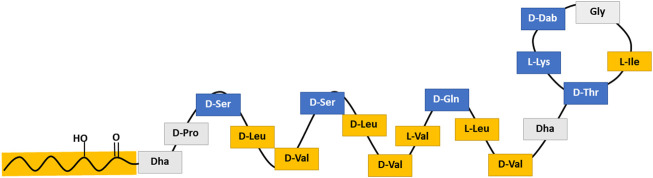
Schematic structure of tolaasin II representing the amphipathic α-helical golf-club conformation assumed for tolaasin II. Blue stands for amino acids with low hydrophobicity, yellow for those with high hydrophobicity and gray for those with a hydrophobicity value close to zero according to the scale of Kyte and Doolittle (Kyte and Doolittle, 1982).

The cyclic lipopeptide tolaasin II was produced by *Pseudomonas tolaasii* CH36 (bacterial growth conditions as described in literature (Bassarello et al., 2004)). Figure 1 schematically shows the amphiphilic *α*-helical golf-club conformation of tolaasin II in DPC/water. Tolaasin II differs in one amino acid (Gly^16^ instead of Hse^16^) from tolaasin I. In short, produced metabolites were extracted from a bacterial broth by overnight precipitation by the addition of 150 g/L CaCl_2_. The residue was then separated by centrifugation. Subsequently, tolaasin II was isolated and purified *via* preparative HPLC. Purification was performed by injection into a Prostar HPLC device (Agilent Technologies) equipped with a Luna C-18 (2) preparative RP-HPLC column (250 × 21.2 mm, 5 μm particle size). An optimized elution gradient of H_2_O/CH_3_CN was applied at a flow rate of 17.5 ml min^−1^, while the column temperature was kept at 35°C. The purified compound was then characterized and quantified by liquid state NMR spectroscopy. All NMR measurements were performed on either a Bruker Avance III spectrometer operating at a respective ^1^H and ^13^C frequency of 500.13 MHz and 125.76 MHz and equipped with a BBI-Z probe or a Bruker Avance II spectrometer operating at a ^1^H and ^13^C frequency of 700.13 MHz and 176.05 MHz, respectively, and equipped with a 5 mm Prodigy TCI probe. The sample temperature was set to 298.0 K. Standard pulse sequences as present in the Bruker library were used throughout. High precision 5 mm NMR tubes (Norell, Landisville, NJ) were used. Dimethylsulfoxide-d6 (DMSO-d6) (99.50%) was used as solvent throughout and was purchased from Eurisotop (Saint-Aubin, France). ^1^H and ^13^C chemical shift scales were calibrated by using the residual solvent signal using TMS as secondary reference. Quantification was done by using ERETIC methodology based on PULCON as described by Wider and Dreier (Wider and Dreier, 2006). Details on characterization and purity can be found in the Supplementary Material. Tolaasin II was shipped and stored as dry powder at −20°C. It was first solubilized in DMSO to a 20 mM stock solution and further diluted with Tris buffer to respective concentrations before further usage in experiments.

### Liposome preparation and characterization

Calcein loaded large unilamellar liposomes (calcein-LUVs) were produced with either 14:1 PC, 16:1 PC, POPC, 18:1 PC, or 20:1 PC. The lipids were dissolved in chloroform, which was subsequently evaporated at 30–40 mbar and 1°C in a desiccator connected to a vacuum pump for thin lipid film preparation. The chloroform of the POPC containing solution was evaporated with a vacuum centrifuge at 36°C. All lipid films were placed under high vacuum overnight. Enclosed air in the vial was exchanged for inert argon and vials were sealed with parafilm. Lipid films were stored at –20°C or immediately used for liposome preparation.

Lipid films were then hydrated with calcein-Tris buffer (70 mM calcein, 10 mM Tris, 0.5 mM EDTA, pH 7.4) at room temperature. Hydration was accompanied by four freeze-thaw cycles with dry ice and a water bath set to 50°C.

The lipid dispersion was extruded with a LIPEX™ Thermobarrel Extruder (Evonik Industries AG, Essen, Germany) under high pressure through two stacked 100 nm filters (Whatman^®^ Nuclepore™). Not encapsulated, free calcein was removed by size exclusion chromatography (PD-10 desalting column from GE Healthcare, Little Chalfont, United Kingdom) and thus the external calcein-Tris buffer replaced by iso-osmotic Tris buffer (10 mM Tris, 110 mM NaCl, 0.5 mM EDTA, pH 7.4).

Calcein-LUVs were characterized by their hydrodynamic diameter (Z-average) and their sample homogeneity with dynamic light scattering (Zetasizer Nano ZS, Malvern Panalytical Ltd, Worcestershire, United Kingdom). In addition to this, the lipid concentration of each calcein-LUVs batch was analyzed according to the Bartlett Assay (Bartlett, 1959). Calcein-LUVs were stored at room temperature to avoid phase transition.

### Calcein leakage assay by TCSPC

Calcein-LUVs in concentrations of 30 μM, 60 μM, 100 μM, 200 μM, and 300 µM (14:1 PC—20:1 PC, or POPC) were added to tolaasin II solutions in Tris buffer with respective membrane-active concentrations. All samples were incubated in disposable fluorescence cuvettes (Sarstedt AG and Co. KG, Nümbrecht, Germany) on a rotary shaker with 400 rpm at 25°C under the exclusion of light for 1 h. Samples containing only calcein-LUVs (14:1 PC—20:1 PC, or POPC) and Tris buffer were always carried along as controls. Tolaasin II triggered membrane permeabilization (calcein leakage) was then measured with a FluoTime 100 spectrometer (PicoQuant, Berlin, Germany) by time-correlated single-photon counting (TCSPC). Right before fluorescence data acquisition, all samples incubated at more than 30 µM lipid were diluted to a common lipid concentration of 30 µM (and, hence, a common total calcein concentration) by addition of Tris buffer (Fiedler and Heerklotz, 2015; Steigenberger et al., 2021).

The fluorescent dye calcein was excited at 467 nm by a pulsed laser diode (LDH-P-C-470, PicoQuant, Berlin, Germany) operated by a PDL 800-D laser driver (pulse width: 20 ps; repetition rate: 20 MHz). The attenuators were set to ensure a detection rate of <1% photons to avoid pile-up effects. Emission was detected at >530 nm (OG530 longpass filter included) by a PMA 175-N detector (PicoQuant, Berlin, Germany). Lifetime-decays were acquired for 30 s (resolution of 25 ps per bin). A Ludox HS-40 scattering standard was used to detect the instrument response function to fit the data by a reconvolution fit with PicoQuant’s FluoFit software (Berlin, Germany).

Calcein is a self-quenching fluorophore and its fluorescence lifetime strongly correlates with its local concentration. Samples containing only intact liposomes with an interior calcein concentration of 70 mM should display a fluorescence lifetime of τ_E_ ≈ 0.4 ns. Samples in which all calcein was released from the liposome interior contain a final calcein concentration of about 5 µM and should display the lifetime of dilute, free dye τ_F_ ≈ 4 ns. Liposomes having released some of the originally entrapped dye give rise to entrapped calcein populations of 70–0 mM, fluorescing with intermediate lifetimes τ_E_ (subscript E for entrapped) between 0.4 and 4 ns. A free biexponential (Eq. 1) fit model was chosen for data analysis. This is a significant simplification of the actual sample composition as not all liposomes in one sample leak the same amount of calcein. However, it yielded the most stable fits with a rather good separation between free and entrapped dye as long as τ_E_ is well below 4 ns (optimally ≤1 ns).

$$B\left(t\right)=B_{E}\cdot e^{-\frac{t}{\tau_{E}}}+B_{F}\cdot e^{-\frac{t}{\tau_{F}}}$$ (1)

B(t) represents the frequency of detecting photons at a certain time after excitation (ns), B_E_ and B_F_ represent the proportion of entrapped (subscript E) and free (subscript F) calcein. Respectively, τ_E_ and τ_F_ represent the corresponding fluorescence lifetimes. The fraction of calcein having leaked out (L) can then be quantified by Equation 2.

$$L=\frac{B_{F}-B_{F0}}{B_{F}-B_{F0}+Q_{Stat}\cdot B_{E}}$$ (2)

B_F0_ corrects for incomplete free calcein removal by size exclusion chromatography and for the intrinsic leakiness of the respective calcein-LUVs in the absence of CLiP. It is represented by the controls to which no tolaasin II was added. Quenching of calcein fluorescence is almost exclusively collisional, the Stern-Volmer constant of dynamic quenching, K_D_ ≈ 0.13 mM^−1^ is much larger than that of static quenching, K_S_ ≈ 0.005 mM^−1^ (Patel et al., 2009). The empiric factor Q_Stat_ = 1.2 in Equation 2 corrects for these small effects. For more details on the theoretical background of the calcein leakage assay, please refer to the literature (Patel et al., 2009; Fiedler and Heerklotz, 2015; Stulz et al., 2019; Steigenberger et al., 2021).

### Equi-activity analysis

Data were analyzed with an equi-activity analysis as described in detail in literature (Encinas and Lissi, 1982; de la Maza and Parra, 1997; Heerklotz and Seelig, 2007; Patel et al., 2009; Fan et al., 2014; Steigenberger et al., 2021). In brief, membrane leakage, L, was displayed as a function of the respective peptide concentration, c_P_, for all tested lipid concentrations, c_L_, (30 µM–300 µM) in one graph (see Supplementary Material). Subsequently, the necessary c_P_ triggering a certain L at a specific c_L_ can be read off the graph by interpolation. Taking into account that the total peptide concentration, c_P_, comprises the membrane bound (c_P_^b^) and free (c_P_^aq^) fractions (all concentrations referring to total sample volume) and defining the mole ratio of membrane-bound peptide per lipid, R_e_ ≡ c_P_^b^/c_L_, we obtain:

$$c_{P}\left(L\right)=R_{e}\left(L\right)\cdot c_{L}+{c_{P}}^{aq}\left(L\right)$$ (3)

That means, plotting the interpolated peptide concentrations, c_P_(L), needed to cause a certain leakage, L, obtained by measurements at different lipid concentration, c_L_, as a function of this lipid concentration yields a straight line with the slope R_e_ and the *Y*-intercept c_P_^aq^. These parameters represent the membrane composition leading to the leakage L and the aqueous CLiP concentration that is in equilibrium with this membrane. c_P_^aq^ is linked to R_e_ *via* the apparent partition coefficient, K:

$$K=\frac{{c_{P}}^{b}}{c_{L}\cdot {c_{P}}^{aq}}=\frac{R_{e}}{{c_{P}}^{aq}}$$ (4)

Finally, repeating this procedure to derive R_e_(L) and c_P_^aq^(L) for different leakage values, L, provides the universal leakage curve solely depending on the intrinsic membrane permeabilizing activity of membrane bound CLiP, L(R_e_), and the partition isotherm, c_P_^aq^(R_e_). K^−1^ can be understood as a dissociation constant in a concentration unit, which was chosen for data presentation.

It should be noted that the interpolation procedure can induce a smoothening of R_e_(L) and c_P_^aq^(L) if more L levels are evaluated than actual points measured in the sensitive L range, about 20%–80%, on each curve. In particular for partitioning isotherms to be fitted with statistical error analysis, points should be limited to mutually independent ones by picking L values in close proximity to actual leakage data points.

The standard assay testing different peptide concentrations at a single lipid concentration is usually done in triplicates. In the equi-activity analysis as performed here, each CLiP concentration is tested 5 times, but at different lipid concentrations. The statistical information from the standard deviation of three identical samples in the standard assay is now obtained from the deviations of five points from linearity of the fit according to Equation 3. This way, the equi-activity analysis provides additional information on R_e_(L) and K without compromising statistical significance and with only little more peptide and experimental time needed.

### Kinetics of membrane permeabilization

Tolaasin II-triggered membrane permeabilization of all calcein-LUVs (14:1 PC—20:1 PC, or POPC) was monitored over a time period of 24 h. The first measurement was done immediately after addition of the calcein-LUVs to the tolaasin II solution, with the detection accumulating over 30 s. More data points were taken after 10, 30, 60, 120, and 1,440 min. Additionally, leakage of a further experimental series with POPC calcein-LUVs was monitored over a time period of up to 72 h.

All leakage samples contained 30 µM of the respective calcein-LUVs and were incubated on a rotary shaker at 400 rpm at 25°C and protected from light. Membrane permeabilization was measured by TCSPC as described.

## Results

### Kinetics of membrane permeabilization

**FIGURE 2:**
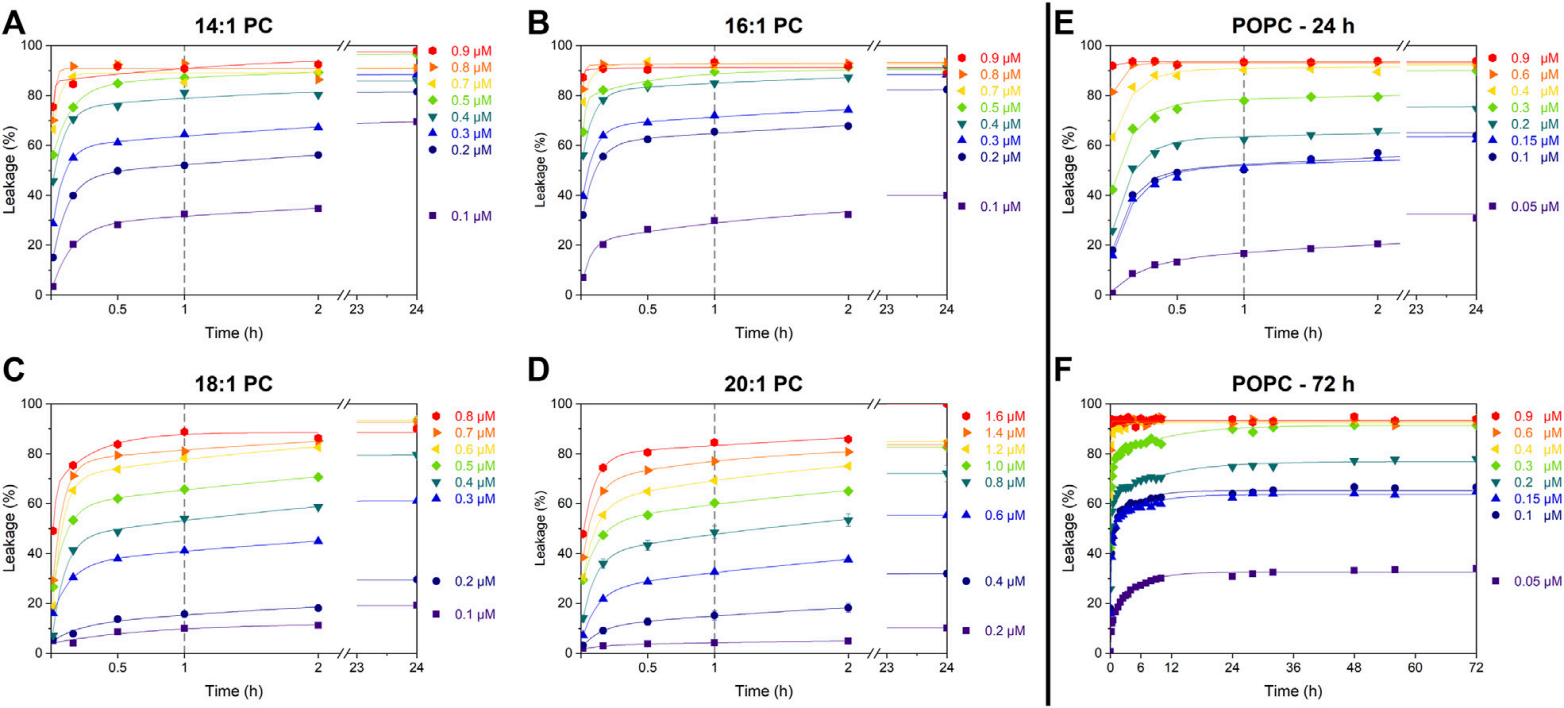
Cumulative leakage kinetics of 14:1 PC **(A)**, 16:1 PC **(B)**, 18:1 PC **(C)**, 20:1 PC **(D)**, and POPC **(E and F)** at 30 µM lipid concentration. Data points depict calcein leakage (%) as a function of time for different tolaasin concentrations as indicated next to each plot. Lines are to guide the eye only. The vertical gray dashed line after 1 h represents the standard incubation time; the fast leakage component appears to be essentially completed by this time.

Cumulative leakage data depict the progression of membrane permeabilization as a function of time. They may help to distinguish between different leakage mechanisms–such as asymmetry stress or formation of disordered toroidal or barrel-stave like pores–acting in parallel. To this end, calcein leakage of all tested calcein-LUVs (at 30 µM lipid) was measured over at least 24 h (Figures 2A–E) at different concentrations of tolaasin II (hereafter simply referred to as tolaasin).

All data sets follow the well-known pattern (Wimley and Hristova, 2019) previously assigned to a combination of two leakage mechanisms with distinct kinetics. The first, fast but limited mechanism proceeds over some minutes, until the defects anneal and the leakage by this mechanism stops. The fraction of dye leaking by this mechanism increases with CLiP concentration. This behavior has previously been assigned to asymmetry-stress driven, transient leakage (Guha et al., 2019; Wimley and Hristova, 2019).

The second, slow process proceeds over many hours to days and may ultimately proceed to 100% leakage–the data demonstrate this for the highest peptide concentrations used but do not reveal this in detail for lower concentrations of tolaasin II. This behavior is expected, for example, for toroidal or barrel-stave pores. A low likelihood of forming a pore causes slow leakage but once leakage occurs, it should continue as pores reappear with the same likelihood. Given the scope of this study, we abstained from fitting a kinetic model (Heerklotz and Seelig, 2007) to the curves. Crucially, tolaasin induces transient, limited leakage in all membranes, regardless of their thickness (Figure 2). The leakage after the standard incubation time of 1 h primarily represents the extent of this transient leakage mechanism. Under the conditions of these experiments, a detailed quantitative characterization of the slow leakage mechanism does not appear to be warranted.

### Dose-response curves at low and high lipid concentration

**FIGURE 3:**
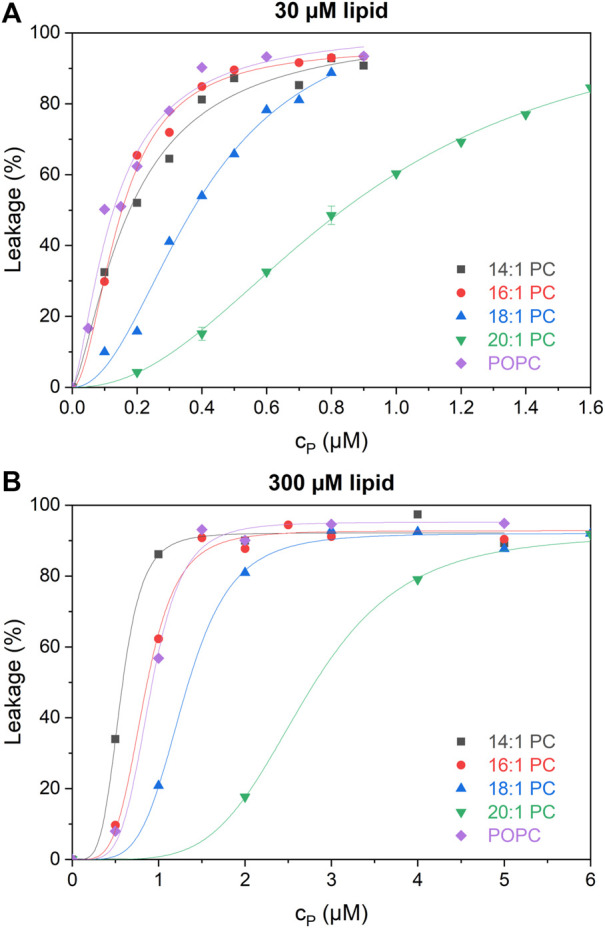
Calcein leakage L as a function of total tolaasin concentration c_P_ (µM) after 1-h incubation time in 30 µM **(A)** and 300 µM **(B)** of the tested lipids (see color code in the figure). Lines to guide the eye are fitted by the Hill equation.

Figure 3 shows dose-response curves of leakage induced by tolaasin after 1 h of incubation with liposomes of the different lipids at 30 µM (panel A) and 300 µM (panel B). Tolaasin is highly active in leaking in particular membranes composed of shorter-chain lipid and POPC. In this case, tolaasin concentrations causing 50% leakage after 1 h, c_P_^50^, are of the order of 0.1 µM for 30 µM lipid. Significantly higher tolaasin concentrations are needed to leak the long-chain lipid bilayers, particularly those of 20:1 PC.

At 300 µM lipid, c_P_^50^ increases monotonically with chain length for the symmetric-chain lipids from 14:1 through 20:1 PC. This order is changed for 30 µM lipid, where 16:1 PC is at least as susceptible to leakage as 14:1 PC. This change in order suggests a switch from a low-lipid or intermediate-lipid regime at 30 µM lipid, where little peptide is bound and partitioning limits activity, to a high-lipid regime at 300 µM (Steigenberger et al., 2022). A detailed, quantitative analysis of this phenomenon is pursued in the following using an equi-activity analysis, allowing to distinguish between partitioning effects and intrinsic activity of membrane-bound CLiP quantitatively.

### Equi-activity analysis

**FIGURE 4:**
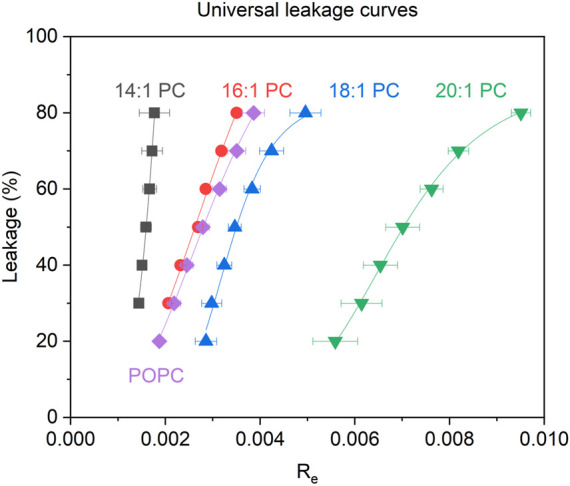
Calcein leakage L after 1 h as a function of the membrane-bound tolaasin to lipid mole ratio (R_e_) for LUVs consisting of the tested lipids (see color code in the figure) triggered by tolaasin. Errors of R_e_ are standard errors of the linear regression of the equi-activity lines (see Supplementary Material).

All leakage data used for the equi-activity analysis as well as the respective equi-activity plots and linear regression data are displayed in the Supplementary Material. A detailed explanation on how the equi-activity analyses are performed is described in literature (Steigenberger et al., 2021; Steigenberger et al., 2022). This analysis provides universal leakage curves (Figure 4), which are generated by plotting membrane leakage after 1 h as a function of the local content of membrane-inserted peptide per lipid in the membrane (mole ratio R_e_).

Figure 4 shows the universal leakage curves of tolaasin for the different lipids studied. For a typical large unilamellar vesicle consisting of about 10^5^ lipid molecules, R_e_^50^ = 0.0016 implies that approximately 160 tolaasin molecules per liposome are needed to produce 50% of calcein leakage in 14:1 PC liposomes. With increasing chain length of the lipids from 14:1 to 20:1, higher tolaasin contents are required in the membrane to cause the same extent of leakage. For 20:1 PC, R_e_^50^ corresponds to about 700 tolaasin molecules per liposome of 10^5^ lipids.

Table 1 lists the values of R_e_^50^ along with the hydrophobic bilayer thickness, d(_l_), of the different membranes according to the literature. In summary, the universal dose-response curves suggest that thicker lipid bilayers are more resistant to the intrinsic membrane permeabilizing activity of tolaasin.

**TABLE 1:** The mole ratio of membrane-bound tolaasin molecules per membrane lipid needed to trigger 50% calcein leakage (R_e_^50^) after 1 h and the hydrophobic bilayer thickness, d_l_, as published by Kucerka et al. (2008), Kucerka et al. (2009) for the lipids studied.

Lipid	R_e_^50^	d_l_ (nm)^a^
**14:1 PC**	0.0016	3.37
**16:1 PC**	0.0027	3.62
**18:1 PC**	0.0035	3.87
**20:1 PC**	0.0070	4.25
**POPC**	0.0028	3.68

^a^ Kucerka et al. (2008), Kucerka et al. (2009)

**FIGURE 5:**
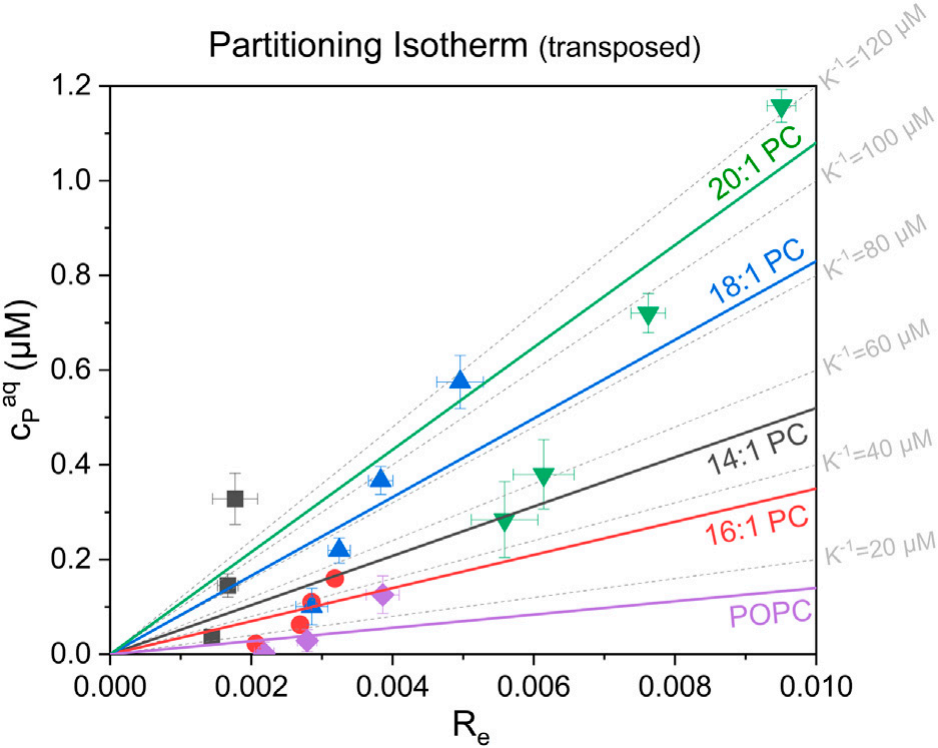
Transposed partitioning (i.e., “dissociation”) isotherms showing the respective free, aqueous tolaasin concentration, c_P_^aq^ (µM) as a function of the equilibrium tolaasin-to-lipid mole ratio within the membrane, R_e_, for the different lipids studied (see plot). The error bars are standard errors of R_e_ and c_P_^aq^ from the linear fit of the equi-activity analysis. The colored lines represent instrumental-error-weighted, linear fits of the “dissociation isotherms” for each tested lipid (see color code in figure). Their slope represents the apparent membrane dissociation constant (i.e., reciprocal apparent membrane-buffer partition coefficient), K^−1^ for tolaasin with the tested lipids (see color code in the figure). Grey dotted grid lines display fictive isotherms for selected K^−1^ values.

Aside from the universal leakage curves, the equi-activity plots (see Supplementary Material) also allow, in principle, to calculate the apparent partitioning coefficient K (see Eq. 4) of a membrane-active compound between membrane (R_e_) and aqueous solution (c_P_^aq^) and thus, a characterization of its partitioning behavior. However, whereas the determination of R_e_ is very robust and accurate even at very low active concentrations, the determination of c_P_^aq^ becomes very error-prone or even impossible for compounds with very high membrane affinity (Fan et al., 2014; Steigenberger et al., 2021) or, as for tolaasin, with very high activity. In both cases, the aqueous concentrations are extremely low at leakage levels of about 20%–80%, so that this analysis only provides a rough estimate of the partitioning behavior. The partitioning isotherms of tolaasin II are shown in transposed form as free versus membrane-inserted CLiP in Figure 5. Please note that tolaasin shows an R_e_ scale one to two orders of magnitude below that described for other membrane-active compounds (typically from 0.02 to 0.55) (Heerklotz and Seelig, 2007; Patel et al., 2011; Fan et al., 2014; Fiedler and Heerklotz, 2015; Steigenberger et al., 2021). Please see Supplementary Material regarding the statistical analysis of the fit. In spite of the problems mentioned, Figure 5 allows for a number of important observations to be made. First, the binding affinity of tolaasin is rather low into all membranes tested here with high K^−1^ values between 10 and 100 µM.

Knowledge of the order of magnitude of K^−1^ is crucial for the interpretation of activity and selectivity as partitioning affects the overall membrane activity differently in the high (c_L_ ≫ K^−1^), intermediate, and low (c_L_ ≪ K^−1^) lipid regimes, respectively (Loffredo et al., 2021; Steigenberger et al., 2022). Virtually complete tolaasin binding will only occur in the high lipid regime with lipid concentrations much greater than K^−1^. This is clearly the case for the established dose-response curves at 300 µM after 1-h incubation. In this case, membrane-activity is determined solely by the intrinsic membrane permeabilizing activity of the peptide (R_e_). This is also the reason for the observed changes in membrane activity in the dose-response curves at 30 μM and 300 µM lipid, which were previously shown in Figure 3. At 300 µM lipid, membrane permeabilizing activity increases clearly in line with R_e_. At 30 µM lipid concentration, partitioning affects the available active peptide concentration in each system to a different extent. Furthermore, Figure 5 suggests partitioning of tolaasin to become weaker with increasing membrane thickness at least from 16:1 PC to 18:1 PC and 20:1 PC. Similar observations have been made for the cationic antimicrobial peptide maculatin 1.1, which also showed highest peptide incorporation into the 16:1 PC membrane (POPC not tested), lower binding to 14:1 PC membranes and a continuous decrease in binding affinity with lipid chains >16 carbons up to 22:1 PC (Lee et al., 2018).

## Discussion

### Thicker membranes reduce leakage activity, also for asymmetry-stress driven leakage

Let us, first, come back briefly to the mode of action of tolaasin in the experiments presented here. As discussed in more detail elsewhere (Steigenberger et al., 2022), the observation of limited leakage for all systems (also in the low-lipid regime where the aqueous solution provides a reservoir for maintaining the peptide content in the outer leaflet) implies the leakage seen here to be mainly asymmetry-stress driven. On its own, this does not rule out other mechanisms, including the formation of oligomeric pores, to happen and potentially dominate under different conditions. In fact, the formation of membrane-spanning pores by the assembly of 6–8 tolaasin molecules, but also a detergent-like action at higher tolaasin concentrations (Cho and Kim, 2003; Jourdan et al., 2003; Coraiola et al., 2006; Jo, 2011) have been discussed as possible mechanisms of action. Andolfi et al. (2008) also hypothesized that tolaasin activity in their experimental setup was caused by peptide monomers rather than peptide oligomers.

**FIGURE 6:**
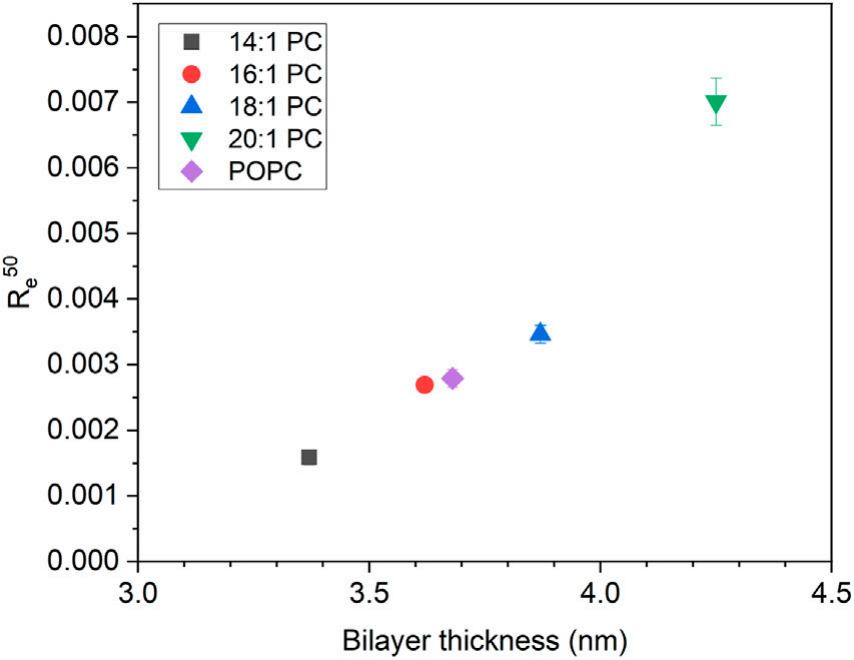
The mole ratio of membrane-bound tolaasin per lipid to cause 50% leakage (R_e_^50^) as a function of hydrophobic bilayer thickness for lipid membranes composed of the tested lipids according to Kucerka et. al. (2008), Kucerka et. al. (2009) (see color/symbol code in the figure). R_e_^50^ errors are standard errors of linear regression of equi-activity analysis.

A key question in the introduction has been whether in this particular case, leakage activity would increase with thickness due to increased monolayer curvature stress and packing mismatch or whether it would decrease as in most other examples because of an increase in membrane stability (see Supplementary Material for a schematic representation of tolaasin-induced monolayer curvature stress in thicker or thinner membranes). The universal leakage curves in Figure 4 and the related Figure 6, showing the mole ratio of membrane-bound tolaasin to cause 50% leakage plotted *versus* hydrophobic membrane thickness, support the second hypothesis. Given that thickness may correlate with several membrane properties such as order or bending stiffness, caution is advised in identifying one of these membrane properties to be primarily causal for the leakage inhibition.

Hence, the expectation of curvature stress to increase with membrane thickness is not supported by the onset of leakage data. Rather it appears to be overcompensated by an increase in membrane stability. However, it can be assumed to account for the possible decrease of membrane affinity (increase of K^−1^) with increasing membrane thickness. As demonstrated in detail for detergents (Heerklotz and Seelig, 2001; Heerklotz, 2008), the main parameter governing K^−1^ of an amphiphile is the hydrophobic surface area that becomes screened from water upon membrane insertion. This is a property of the peptide and rather not of the membrane. For the example of detergents of the type C_12_EO_n_, this is given by the common dodecyl chain and causes a roughly tenfold lower K^−1^ than for corresponding C_10_EO_n_ compounds. However, increasing curvature stress with increasing headgroup size causes a consistent tenfold increase of K^−1^ as “n” increases from 3 to 8 (Heerklotz and Seelig, 2001).

Literature data, for example on gramicidin S (Ashrafuzzaman et al., 2008), showing little or no decrease in leakage activity with increasing membrane thickness, may also indicate that the overall thickness effect is governed by the balance of two opposing phenomena. These two opposing phenomena are, first, increased overall stability (opposing leakage) and, second, hydrophobic-mismatch induced curvature strain (expected to promote leakage).

### Implications for antimicrobial activity

There have been interesting qualitative and quantitative parallels between liposome leakage data and antimicrobial activity (Fiedler and Heerklotz, 2015; Stulz et al., 2019; Steigenberger et al., 2021) which suggest that unspecific membrane permeabilization is at least one mode of action of antimicrobial peptides. Of course, this does not rule out a specific action of a peptide on protein-based signaling or function, perhaps with active membrane permeation providing a means of delivery of the peptide to its final site of action. Comparison of liposome and *in vivo* data requires proper handling of the crucial lipids and the effective lipid concentration (deciding whether an experiment is done in high- or low-lipid regime (Loffredo et al., 2021; Steigenberger et al., 2022)) and consideration of the effects of compartment size (Braun et al., 2018) on the absolute and relative extent of leakage. Furthermore, the truly relevant membrane leakage mechanism might be specific to a certain cargo and/or focused on specific membranes or membrane nano-environments. Versatile modes of action of AMPs are also discussed as a possible reason for the low development of resistance in nature, although they are ubiquitously present (Peschel and Sahl, 2006).

There is yet no firm explanation on how tolaasin kills target cells at the molecular level. The wide variety of possible leakage mechanisms described in the literature raises the question whether tolaasin actually exerts its membrane activity *via* a combination of several modes of action. Moreover, the primary mode of action exploited by the peptide could be adapted to properties of the target organism. As a consequence of leakage, it is conceivable that tolaasin action *in vivo* disturbs the electrochemical potential or the osmotic gradient, thereby causing decompartmentalization of a cell or cell compound, or complete cell lysis. This is believed to trigger tyrosinase activation and subsequently increased melatonin and quinone production, responsible for the observed pitting and browning of brown blotch disease, causing large economic losses (Soler-Rivas et al., 1999).

Both fungal and plant membranes contain specific sterols and sphingolipids and have been reported to comprise different domains or regions that must be considered to differ in thickness and order (Cacas et al., 2016; Gronnier et al., 2016; Athanasopoulos et al., 2019; Santos et al., 2020). Our data suggest that the thinner regions of these membranes might be more susceptible to the permeabilizing action of tolaasin and similar CLiPs. Lateral membrane heterogeneities could also play a crucial role. For the *Bacillus* CLiP surfactin, for example, it was shown that it inserts preferentially at the boundaries between the gel and liquid phases of model membranes (Deleu et al., 2013; Gilliard et al., 2022). However, effects of sterols may be much more specific than on order and thickness only. For example, studies reported a stronger inhibition of tolaasin I and fengycin activity in membranes containing cholesterol than in membranes containing ergosterol (Fiedler and Heerklotz, 2015; Ferrarini et al., 2022). The extent of surfactin leakage was independent of both ergosterol and cholesterol added to POPC (Fiedler and Heerklotz, 2015) but cholesterol slowed down leakage kinetics in the first few seconds after mixing (Carrillo et al., 2003).

A biological organism can regulate its membrane composition to some extent to respond to external conditions. Classic papers in this respect have described the regulation of membrane lipid composition with environmental conditions for *Acholeplasma laidlawii* (Lindblom et al., 1993) and *Escherichia coli* (Rietveld et al., 1993) and polymyxin resistance in bacteria by *mcr-1* phenotypes (Khondker et al., 2019). Hence, changing the thickness of the hydrophobic core could be a mechanism of resistance of a microbe against a membrane permeabilizing agent, just as it is already known for altering membrane order (Tran et al., 2015; Assoni et al., 2020).

## Conclusion

Tolaasin is a very potent permeabilizing agent for PC membranes with typical active concentrations of the order of 0.1 µM. This activity arises from a very strong intrinsic membrane permeabilizing activity (R_e_^50^ as low as 0.3 mol% within the membrane), sensitive to membrane thickness. Tolaasin shows a monotonically decreasing local activity (increasing R_e_^50^) with increasing membrane thickness (hydrophobic core of bilayer ranging from about 3.4 through 4.3 nm). This effect is opposed by rather weak partitioning with “dissociation constants” K^−1^ of the order of 10–100 µM. The finding of limited leakage over a time course of minutes also in the low-lipid regime implies the mode of action observed here to be based on asymmetry-stress induced membrane failure. The formation of oligomeric barrel-stave pores that are too short to easily span the thicker membranes could also explain the thickness effect but not the limited leakage. Thicker membranes should suffer stronger packing mismatch and spontaneous monolayer curvature with the peptides than thinner ones, which is expected to lower the activation energy for a transient defect to form. However, this tendency is likely compensated by an opposing increase in overall membrane stability with increasing thickness.

Overall, membrane thickness and mechanical stability are parameters that differ between biological membranes and can be tuned to some extent by an organism. Hence, they must be considered to contribute to selectivity and resistance mechanisms.

In the standard assay to check for membrane thickness effects as used here, membrane thickness is directly related to order and rigidity so that this approach cannot truly distinguish which of these properties primarily controls the membrane affinity and the intrinsic membrane activity of a membrane-active peptide. Future studies should attempt to study the effects of these membrane properties one at a time. For example, mixtures with different proportions of POPC and DPPC should differ systematically in order but only very little in thickness. Finally, in addition to monitoring the effects of generic membrane properties (such as thickness), it is crucial to consider more specific effects of the lipids and sterols present in fungal, plant or mammalian membranes (Gronnier et al., 2016; Santos et al., 2020). All this would also help to understand the effects of cholesterol *versus* ergosterol on CLiP activity, which is a prerequisite for designing antifungals for medical applications.

## Data Availability

The original contributions presented in the study are included in the article/Supplementary Material, further inquiries can be directed to the corresponding authors.
