# Supplementary material for: The effect of membrane thickness on the membrane permeabilizing activity of the cyclic lipopeptide tolaasin II
Source: Front Mol Biosci. 2022 Dec 23;9:1064742. doi: 10.3389/fmolb.2022.1064742 (PMC9817028; doi:10.3389/fmolb.2022.1064742)
Supplement: Supplementary file 1 [file DataSheet2.pdf]

The effect of membrane thickness on the membrane permeabilizing activity of the cyclic lipopeptide tolaasin II

Jessica Steigenberger, Catherine Mergen, Vic de Roo, Niels Geudens, José C. Martins and Heiko Heerklotz

Supplementary Material

Table of content

1 Purification and characterization of tolaasin II 2
2 Equi-activity analysis 4
2.1 14:1 PC LUVs 4
2.2 16:1 PC LUVs 6
2.3 18:1 PC LUVs 7
2.4 20:1 PC LUVs 9
2.5 POPC LUVs 10
3 Lifetime plot 12
4 Schematic representation of tolaasin-induced monolayer curvature stress 13
5 References 14

1 Purification and characterization of tolaasin II

After 24h incubation of *Pseudomonas tolaasii* CH36 in KB medium, secondary metabolites were extracted by precipitation via salting out. After centrifugation, the precipitate was purified using preparative HPLC chromatography. The tolaasin II eluted at 18.7 minutes.

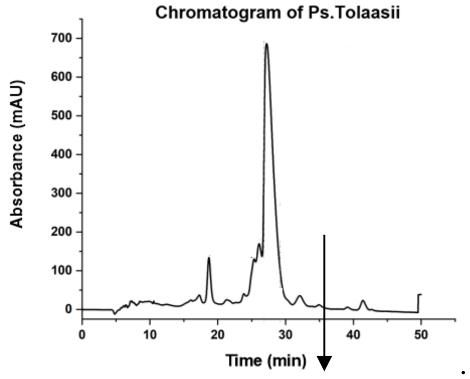

Figure 1.1: Preparative HPLC chromatogram of the extract from *Pseudomonas tolaasii* CH36. The compound of interest, tolaasin II, eluted at 18.7 minutes. The mobile phase composition changed linearly from 0% to 50% AcN in 12 minutes, then 50%→60% AcN in 5 minutes, then 60%→100% AcN in 30 minutes. Finally, 100% AcN was maintained for an additional 3 minutes

The tolaasin II fraction was further analyzed using MS spectrometry and NMR spectroscopy. MS analysis revealed the presence of a single mass of 1943 Da $[M+H]^+$ indicating a molecular formula of $C_{92}H_{159}N_{21}O_{24}$.

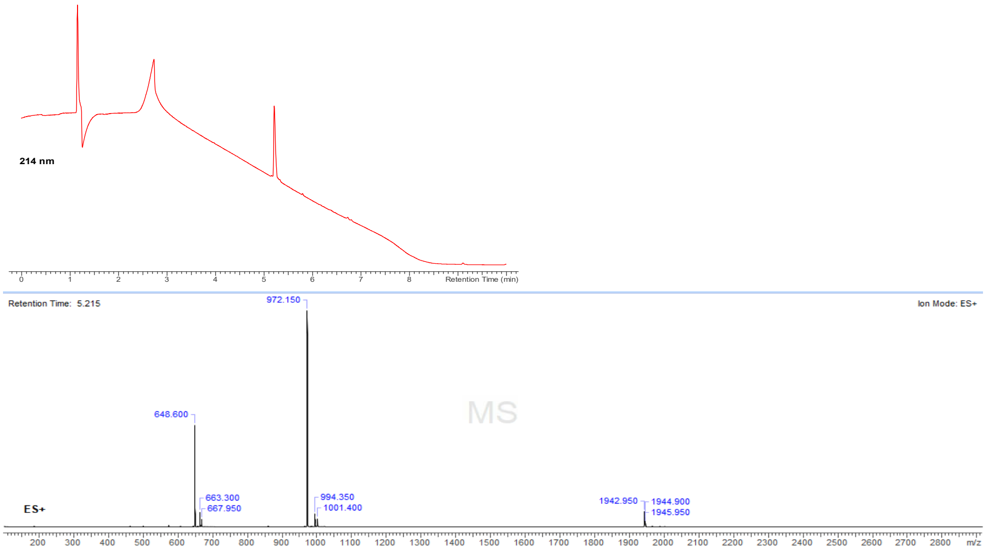

Figure 1.2: LC-MS chromatogram of pure tolaasin II.

Subsequently, tolaasin II was characterized and quantified by liquid state NMR spectroscopy. The NMR chemical shift data corresponds very well to those previous reported[1], indicated that their structure (amino acid sequence and stereochemistry) are identical. Results are listed in Table 1.1.

Table 1.1: Chemical shift data (^{1}H and ^{13}C) of tolaasin II in DMSO-d6 (700MHz, 298K)

		δH / ppm	δC / ppm
**HOA**	C’	-	171.1
	CHβ	3.87	67.4
**ΔBut1**	NH	9.84	-
	C’	-	167.3
	Cα	-	n.d.
	CHβ	5.64	n.d.
	CH3γ	1.7	12.5
**D-Pro2**	NH	n.d.	-
	C’	-	172.5
	CHα	4.24	61.5
	CH2β	2.22	32.2
	CH2γ	1.89/1.80	28.1
	CH2δ	3.59/3.46	38.2
**D-Ser3**	NH	7.84	-
	C’	-	170.6
	CHα	4.2	57.2
	CH2β	3.73	61.5
**D-Leu4**	NH	7.59	-
	C’	-	172.5
	CHα	4.15	52.1
	CH2β	1.49/1.44	40.8
	CHγ	1.55	24.4
	CH3δ	0.76	23.4
	CH3δ	0.76	23.4
**D-Val5**	NH	7.37	-
	C’	-	171.3
	CHα	4.13	58.4
	CHβ	2	29.2
	CH3γ	0.85	18.5
	CH3γ	0.84	18.5
**D-Ser6**	NH	7.8	-
	C’	-	170.4
	CHα	4.32	55.5
	CH2β	3.6	62
**D-Leu7**	NH	7.96	-
	C’	-	172.5
	CHα	4.31	51.5
	CH2β	1.51/1.47	40.9
	CHγ	1.64	23.6
	CH3δ	0.87	23.6
	CH3δ	0.83	21.6
**D-Val8**	NH	7.72	-
	C’	-	171.8
	CHα	4.2	58.7
	CHβ	2	29.1
	CH3γ	0.84	19.7
	CH3γ	0.84	19.7
**L-Val9**	NH	7.84	-
	C’	-	171.6
	CHα	4.18	58.2
	CHβ	2.04	29.1
	CH3γ	0.85	18.1
	CH3γ	0.85	18.1
**D-Gln10**	NH	8.07	-
	C’	-	171.1
	CHα	4.21	53.2
	CH2β	1.89/1.78	28.1
	CH2γ	2.09	31.9
	C(O)NH2δ	7.29/6.80	174
**L-Leu11**	NH	8.05	-
	C’	-	173.7
	CHα	4.41	51.6
	CH2β	1.52	41.1
	CHγ	1.58	23.8
	CH3δ	0.9	18.9
	CH3δ	0.81	18.5
**D-Val12**	NH	8.06	-
	C’	-	170.8
	CHα	4.1	59.1
	CHβ	2.08	31.9
	CH3γ	0.94	19.4
	CH3γ	0.9	18.9
**ΔBut13**	NH	9.34	-
	C’	-	164.5
	Cα	-	n.d.
	CHβ	6.28	n.d.
	CH3γ	1.69	13.3
**D-Thr14**	NH	8	-
	C’	-	171.1
	CHα	4.3	59.3
	CHβ	4.99	71.1
	CH3γ	1.17	17.8
**L-Ile15**	NH	7.87	-
	C’	-	173.6
	CHα	3.77	59
	CHβ	1.63	24.5
	CH2γ	1.56/1.15	n.d.
	CH3γ	0.89	15.5
	CH3δ	0.87	11.4
**Gly16**	NH	9.43	-
	C’	-	n.d
	CHα	3.73/3.68	n.d
**D-Dab17**	NH	7.6	-
	C’	-	170.5
	CHα	4.41	51.4
	CH2β	2.28/2.00	29.1
	CH2γ	2.98/2.91	37.3
	NH2δ	7.82	-
**L-Lys18**	NH	7.61	-
	C’	-	n.d.
	CHα	4.5	50.4
	CH2β	1.52	26.8
	CH2γ	1.28	22.2
	CH2δ	1.85	29.8
	CH2ε	2.71	39.1
	NH2ζ	7.66	-

2 Equi-activity analysis

2.1 14:1 PC LUVs

The dose-response curves of tolaasin II-triggered calcein leakage of 14:1 PC liposomes at lipid concentrations of 30, 60, 100, 200, and 300 µM, respectively, are shown in Figure 2.1, panel A. The total tolaasin II concentrations, $c_{\mathrm{Tolaasin\ II}}$, required to cause a certain degree of calcein leakage (L %) at each lipid concentration, c_{lipid} were read from the corresponding dose-response curves. Horizontal grey lines facilitated read out. Data points are listed in Table 2.1. The thereof created equi-activity plot is shown in panel B of Figure 2.1.

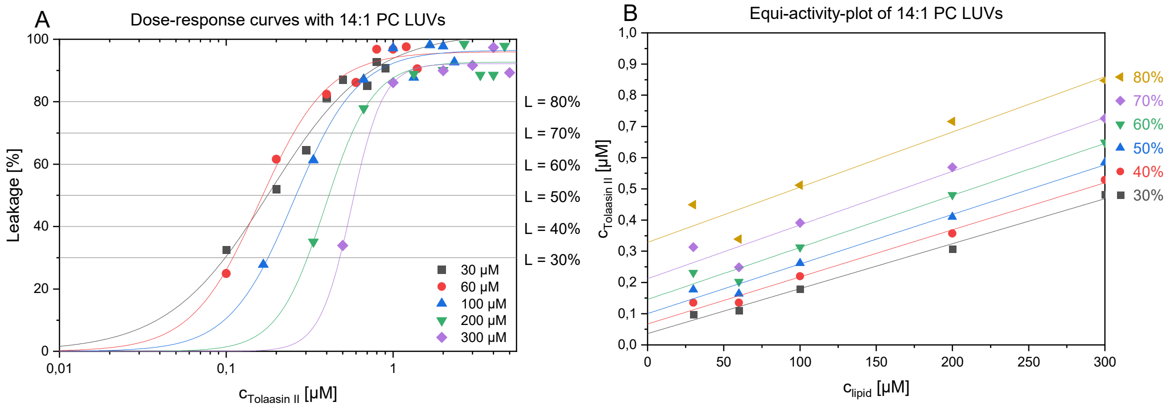

Figure 2.1: Dose-response curves (A) and equi-activity plot (B) with 14:1 PC LUVs. (A) Calcein leakage (%) is shown as a function of total tolaasin II concentration, $c_{\mathrm{Tolaasin\ II}}$, (µM). Leakage data with different lipid concentrations are indicated by color code in the figure. Lines connecting data points are to guide the eye only. Grey horizontal lines are reference lines to indicate 30 to 80% leakage (L) to facilitate data read. (B) $c_{\mathrm{Tolaasin\ II}}$ (µM) is plotted as a function of lipid concentration, c_{lipid} (µM). Colored lines represent fitted equi-activity lines for 30% - 80% leakage, respectively (see color code in figure).

Table 2.1: Data of equi-activity plot for 14:1 PC LUVs. The total tolaasin II concentrations, $c_{\mathrm{Tolaasin\ II}}$, needed to cause a certain degree of calcein leakage (L=X%) at each lipid concentration. Concentrations are given in µM.

$c_{\mathrm{14:1\ PC}}$ (µM)	$c_{\mathrm{Tolaasin\ II}}$ (µM)						
	L=20%	L=30%	L=40%	L=50%	L=60%	L=70%	L=80%
30	-	0.098	0.135	0.177	0.232	0.313	0.449
60	-	0.11	0.135	0.164	0.203	0.249	0.339
100	-	0.179	0.22	0.262	0.313	0.391	0.512
200	-	0.307	0.357	0.41	0.481	0.569	0.716
300	-	0.481	0.529	0.585	0.649	0.726	0.847

A linear regression of each equi-activity line, shown in Figure 2.1, panel B, was performed using the software OriginPro 2019. Table 2.2 presents the fitting data for the y-intercepts (the aqueous tolaasin II concentration, $c_{\mathrm{Tolaasin\ II}}^{\mathrm{aq}}$) and the slope (the effective mole ratio of bound peptide per lipid, R_{e}) with respective standard errors (SD) derived by the linear regression.

For a statistic assessment of the results, it is important to notice that not all fits in, for example, Figure 2.1B are mutually independent. The black points in Figure 2.1B representing 30% leakage arise from the horizontal line at 30% in Figure 2.1A – for each lipid concentration considered, there is an individually measured, experimental data point very close to the 30% level. At 60% leakage, there are experimental data available for 30, 60, and 100 µM, the points for 200 and 300 µM are interpolated between measurements having yielded 30% and 80% leakage, respectively. Hence, we obtain values of $c_{\text{Tolaasin II}}^{aq}$ and R_e for L=30 and 60% that are based on individual, experimental information.

If one now creates another fit at 40%, all points used in Figure 2.1B arise from interpolations from the same experimental data points that yielded the fits at 30% and 60%. Hence, a fit at 40% is based exclusively on interpolated numbers and hence, yields interpolated values for $c_{\text{Tolaasin II}}^{aq}$ and R_e. While this may be useful to generate smooth curves with a meaningful shape, the merely interpolated data do not improve the statistics of the fit and, if considered in error statistics, would result in underestimated errors. For universal leakage curves which are not fitted any further, this provides good, meaningful traces. By contrast, the data of a partitioning isotherm are to be fitted to determine the partition coefficient. For a realistic fit error, these should be limited on points that are essentially based on independent experimental data; these are marked with grey shadings in Table 2.2 and so on.

Table 2.2: Results of equi-activity analysis with 14:1 PC LUVs. Y-intercepts (the aqueous tolaasin II concentration, $c_{\text{Tolaasin II}}^{aq}$) and slope (the effective mole-ratio of bound peptide per lipid, R_e) with respective standard errors (SD) derived by the linear regression of the equi-activity lines shown in Figure 2.1, panel B. Depicted numbers are rounded to four decimal places.

Leakage (%)	$c_{\text{Tolaasin II}}^{aq}$ (µM)	SD $c_{\text{Tolaasin II}}^{aq}$ (µM)	R_e	SD of R_e	R-Square
20	-	-	-	-	-
30	0.0362	0.0137	0.0014	0.0001	0.9875
40	0.0670	0.0157	0.0015	0.0001	0.9853
50	0.1003	0.0197	0.0016	0.0001	0.9791
60	0.1455	0.0248	0.0017	0.0001	0.9700
70	0.2121	0.0371	0.0017	0.0002	0.9386
80	0.3284	0.0542	0.0018	0.0003	0.8818

2.2 16:1 PC LUVs

The dose-response curves of 16:1 PC calcein-LUVs at lipid concentrations of 30, 60, 100, 200, and 300 µM, respectively, are shown in Figure 2.2, panel A. Total tolaasin II concentrations required to cause a certain degree of calcein leakage at each lipid concentration were read from the corresponding dose-response curves. Horizontal grey lines facilitated read out. Data points are listed in Table 2.3. The thereof created equi-activity plot is shown in panel B of Figure 2.2.

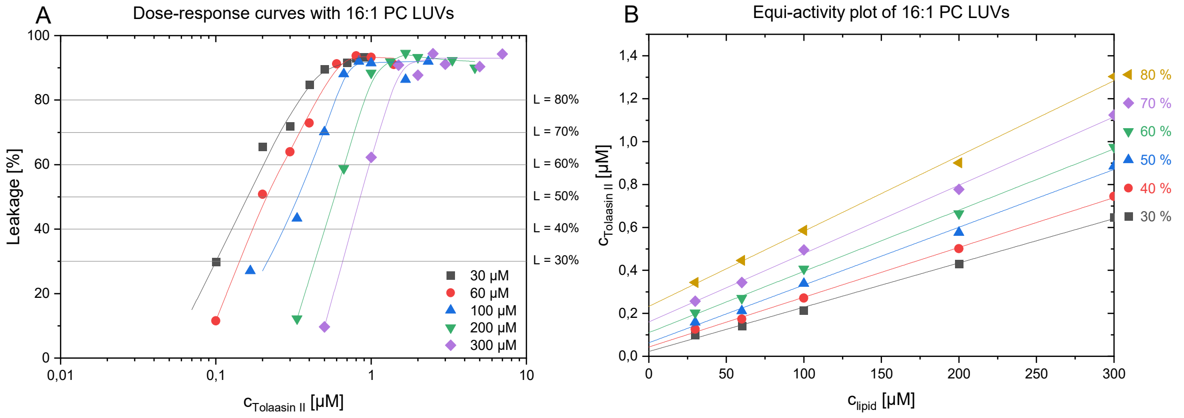

Figure 2.2: Dose-response curves (A) and equi-activity plot (B) with 16:1 PC LUVs. (A) depicts dose-response curves. Calcein leakage (%) is shown as a function of total tolaasin II concentration, $c_{\mathrm{Tolaasin\ II}}$, (µM). Leakage data with different lipid concentrations are indicated by the color code in the figure. Lines connecting data points are to guide the eye only. Grey horizontal lines are reference lines to indicate 30% up to 80% leakage (*L*) to facilitate data read. (B) shows the equi-activity plot. $c_{\mathrm{Tolaasin\ II}}$ (µM) is plotted as a function of lipid concentration, c_{lipid} (µM). Colored lines represent fitted equi-activity lines for 30% - 80% leakage, respectively (see color code in figure).

Table 2.3: Data of equi-activity plot for 16:1 PC LUVs. The total tolaasin II concentrations, $c_{\mathrm{Tolaasin\ II}}$, needed to cause a certain degree of calcein leakage (*L*=X%) at each lipid concentration. Concentrations are given in µM.

$c_{\mathrm{16:1\ PC}}$ (µM)	$c_{\mathrm{Tolaasin\ II}}$ (µM)						
	L=20%	*L*=30%	*L*=40%	*L*=50%	*L*=60%	*L*=70%	*L*=80%
30	-	0.1	0.126	0.159	0.203	0.257	0.344
60	-	0.142	0.173	0.211	0.27	0.344	0.446
100	-	0.214	0.271	0.339	0.407	0.495	0.586
200	-	0.431	0.501	0.577	0.665	0.777	0.901
300	-	0.648	0.745	0.885	0.974	1.124	1.303

A linear regression of each equi-activity line, shown in Figure 2.2, panel B, was performed using the software OriginPro 2019. Table 2.2 presents the fitting data for the y-intercepts (the aqueous tolaasin II concentration, $c_{\mathrm{Tolaasin\ II}}^{\mathrm{aq}}$) and the slope (the effective mole ratio of bound peptide per lipid, R_e) with respective standard errors (SD) derived by the linear regression. Grey shadings highlight the fit data

with the lowest correlation between individual data points from the leakage data set. Only these were considered in the analysis of the partitioning coefficient.

Table 2.4: Results of equi-activity analysis with 16:1 PC LUVs. Y-intercepts (the aqueous tolaasin II concentration, $c_{\text{Tolaasin II}}^{\text{aq}}$) and the slope (the effective mole ratio of bound peptide per lipid, R_e) with respective standard errors (SD) derived by the linear regression of the equi-activity lines of 16:1 PC calcein-LUVs. Depicted numbers are rounded to four decimal places.

Leakage (%)	$c_{\text{Tolaasin II}}^{\text{aq}}$ (µM)	SD of $c_{\text{Tolaasin II}}^{\text{aq}}$ (µM)	R_e	SD of R_e	R-Square
30	0.0222	0.0103	0.0021	0.0001	0.9966
40	0.0430	0.0083	0.0023	0.0000	0.9982
50	0.0628	0.0157	0.0027	0.0001	0.9953
60	0.1102	0.0111	0.0029	0.0001	0.9979
70	0.1601	0.0125	0.0032	0.0001	0.9979
80	0.2323	0.0170	0.0035	0.0001	0.9968

2.3 18:1 PC LUVs

The dose-response curves of 18:1 PC calcein-LUVs at lipid concentrations of 30, 60, 100, 200, and 300 µM, respectively, are shown in Figure 2.3, panel A. Total tolaasin II concentrations required to cause a certain degree of calcein leakage at each lipid concentration were read from the corresponding dose-response curves. Horizontal grey lines facilitated read out. Data points are listed in Table 2.5. The thereof created equi-activity plot is shown in panel B of Figure 2.3.

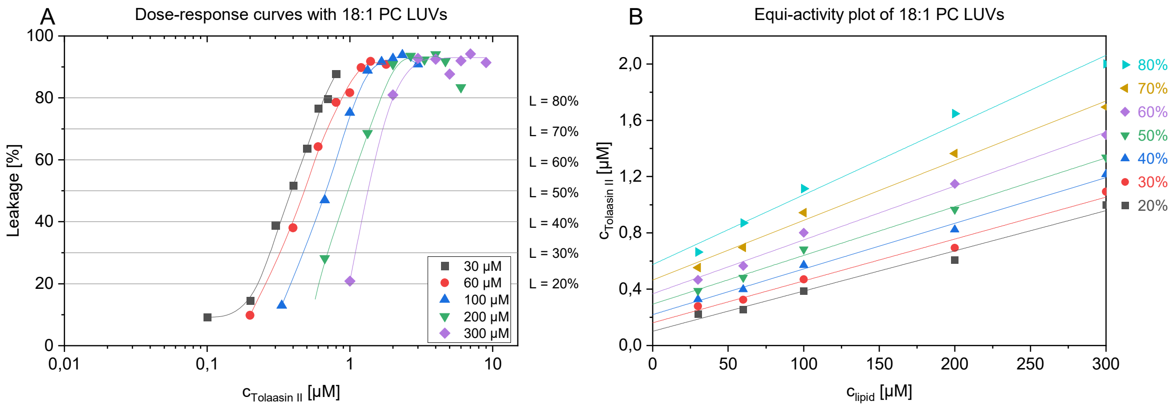

Figure 2.3: Dose-response curves (A) and equi-activity plot (B) with 18:1 PC LUVs. (A) depicts dose-response curves. Calcein leakage (%) is shown as a function of total tolaasin II concentration, $c_{\text{Tolaasin II}}$, (µM). Leakage data with different lipid concentrations are indicated by the color code in the figure. Lines connecting data points are to guide the eye only. Grey horizontal lines are reference lines to indicate 30% up to 80% leakage (L) to facilitate data read. (B) shows the equi-activity plot. $c_{\text{Tolaasin II}}$ (µM) is plotted as a function of lipid concentration, c_{lipid} (µM). Colored lines represent fitted equi-activity lines for 20% - 80% leakage, respectively (see color code in figure).

Table 2.5: Data of equi-activity plot for 18:1 PC LUVs. The total tolaasin II concentrations, $c_{\text{Tolaasin II}}$, needed to cause a certain degree of calcein leakage (L=X%) at each lipid concentration. Concentrations are given in µM.

$c_{\text{18:1 PC}}$ (µM)	$c_{\text{Tolaasin II}}$ (µM)						
	L=20%	L=30%	L=40%	L=50%	L=60%	L=70%	L=80%
30	0.225	0.279	0.328	0.389	0.466	0.553	0.663
60	0.256	0.325	0.398	0.483	0.566	0.697	0.87
100	0.386	0.469	0.57	0.683	0.801	0.944	1.115
200	0.609	0.693	0.823	0.966	1.149	1.364	1.647
300	1.000	1.093	1.216	1.337	1.497	1.694	2.000

A linear regression of each equi-activity line, shown in Figure 2.3, panel B, was performed using the software OriginPro 2019. Table 2.6 presents the fitting data for the y-intercepts (the aqueous tolaasin II concentration, $c_{\text{Tolaasin II}}^{\text{aq}}$) and the slope (the effective mole-ratio of bound peptide per lipid, R_e) with respective standard errors (SD) derived by the linear regression. Grey shadings highlight the fit data with the lowest correlation between individual data points from the leakage data set. Only these were considered in the analysis of the partitioning coefficient.

Table 2.6: Results of equi-activity analysis with 18:1 PC LUVs. Y-intercepts (the aqueous tolaasin II concentration, $c_{\text{Tolaasin II}}^{\text{aq}}$) and the slope (the effective mole ratio of bound peptide per lipid, R_e) with respective standard errors (SD) derived by the linear regression of the equi-activity lines with 18:1 PC calcein-LUVs. Depicted numbers are rounded to four decimal places.

Leakage (%)	$c_{\text{Tolaasin II}}^{\text{aq}}$ (µM)	SD of $c_{\text{Tolaasin II}}^{\text{aq}}$ (µM)	R_e	SD of R_e	R-Square
20	0.1009	0.0383	0.0029	0.0002	0.9756
30	0.1609	0.0361	0.0030	0.0002	0.9799
40	0.2193	0.0268	0.0032	0.0002	0.9907
50	0.2932	0.0230	0.0035	0.0001	0.9940
60	0.3670	0.0294	0.0038	0.0002	0.9919
70	0.4650	0.0431	0.0042	0.0003	0.9859
80	0.5749	0.0559	0.0050	0.0003	0.9826

2.4 20:1 PC LUVs

The dose-response curves of 20:1 PC calcein-LUVs at lipid concentrations of 30, 60, 100, 200, and 300 µM, respectively, are shown in Figure 2.4~~Figure 2.1~~ panel A. Total tolaasin II concentrations required to cause a certain degree of calcein leakage at each lipid concentration were read from the corresponding dose-response curves. Horizontal grey lines facilitated read out. Data points are listed in Table 2.7. The thereof created equi-activity plot is shown in panel B of Figure 2.4.

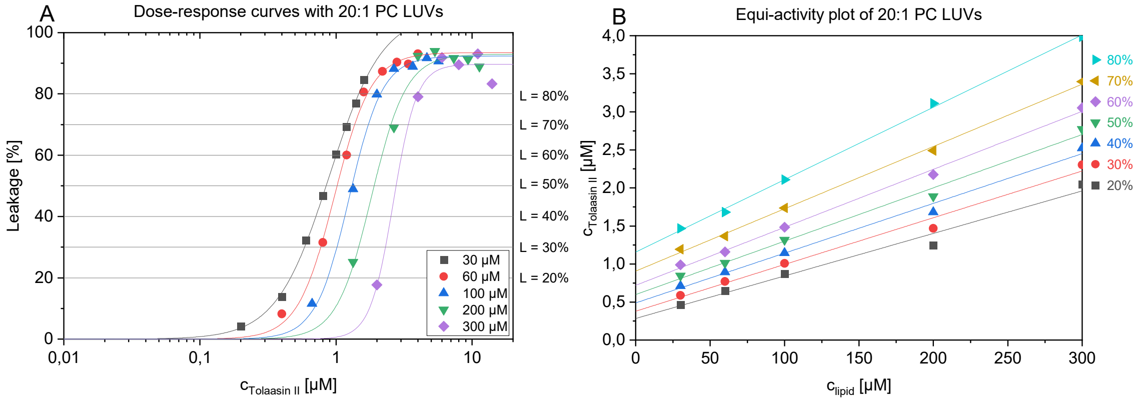

Figure 2.4: Dose-response curves (A) and equi-activity plot (B) with 20:1 PC LUVs. (A) depicts dose-response curves. Calcein leakage (%) is shown as a function of total tolaasin II concentration, $c_{\text{Tolaasin II}}$, (µM). Leakage data with different lipid concentrations are indicated by the color code in the figure. Lines connecting data points are to guide the eye only. Grey horizontal lines are reference lines to indicate ~~30~~% up to 80% leakage (L) to facilitate data read. (B) shows the equi-activity plot. $c_{\text{Tolaasin II}}$ (µM) is plotted as a function of lipid concentration, c_{lipid} (µM). Colored lines represent fitted equi-activity lines for 20% - 80% leakage, respectively (see color code in figure).

Table 2.7: Data of equi-activity plot for 20:1 PC LUVs. The total tolaasin II concentrations, $c_{\text{Tolaasin II}}$, needed to cause a certain degree of calcein leakage (L=X%) at each lipid concentration. Concentrations are given in µM.

$c_{\text{20:1 PC}}$ (µM)	$c_{\text{Tolaasin II}}$ (µM)						
	L=20%	L=30%	L=40%	L=50%	L=60%	L=70%	L=80%
30	0.463	0.587	0.714	0.845	0.99	1.193	1.468
60	0.649	0.769	0.894	1.012	1.158	1.365	1.681
100	0.870	1.008	1.146	1.316	1.484	1.737	2.106
200	1.248	1.468	1.681	1.888	2.176	2.491	3.110
300	2.047	2.304	2.521	2.769	3.051	3.397	3.984

A linear regression of each equi-activity line, shown in Figure 2.4, panel B, was performed using the software OriginPro 2019. Table 2.8 presents the fitting data for the y-intercepts (the aqueous tolaasin II concentration, $c_{\text{Tolaasin II}}^{\text{aq}}$) and the slope (the effective mole-ratio of bound peptide per lipid, R_e) with respective standard errors (SD) derived by the linear regression. Grey shadings highlight the fit data with the lowest correlation between individual data points from the leakage data set. Only these were considered in the analysis of the partitioning coefficient.

Table 2.8: Results of equi-activity analysis with 20:1 PC LUVs. Y-intercepts (the aqueous tolaasin II concentration, $c_{\mathrm{Tolaasin\ II}}^{aq}$) and slope (the effective mole-ratio of bound peptide per lipid, R_e) with respective standard errors (SD) derived by linear regression of the equi-activity lines with 20:1 PC calcein-LUVs. Depicted numbers are rounded to four decimal places.

Leakage (%)	$c_{\mathrm{Tolaasin\ II}}^{aq}$ (µM)	SD of $c_{\mathrm{Tolaasin\ II}}^{aq}$ (µM)	R_e	SD of R_e	R-Square
20	0.2845	0.0801	0.0056	0.0005	0.9721
30	0.3799	0.0733	0.0061	0.0004	0.9806
40	0.4889	0.0615	0.0065	0.0004	0.9879
50	0.5987	0.0607	0.0070	0.0004	0.9897
60	0.7202	0.0413	0.0076	0.0002	0.9959
70	0.9069	0.0366	0.0082	0.0002	0.9972
80	1.1577	0.0346	0.0095	0.0002	0.9982

2.5 POPC LUVs

The dose-response curves of 20:1 PC calcein-LUVs at lipid concentrations of 30, 60, 100, 200, and 300 µM, respectively, are shown in Figure 2.5, panel A. Total tolaasin II concentrations required to cause a certain degree of calcein leakage at each lipid concentration were read from the corresponding dose-response curves. Horizontal grey lines facilitated read out. Data points are listed in Table 2.9. The thereof created equi-activity plot is shown in panel B of Figure 2.5.

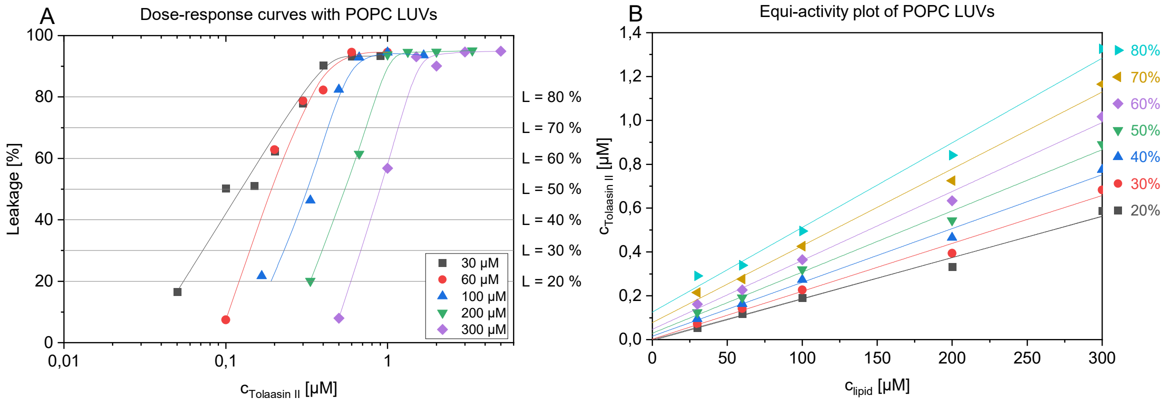

Figure 2.5: Dose-response curves (A) and equi-activity plot (B) with POPC LUVs . (A) depicts dose-response curves. Calcein leakage (%) is shown as a function of total tolaasin II concentration, $c_{\mathrm{Tolaasin\ II}}$, (µM). Leakage data with different lipid concentrations are indicated by color code in the figure. Lines connecting data points are to guide the eye only. Grey horizontal lines are reference lines to indicate 20% up to 80% leakage (L) to facilitate data read. (B) shows the equi-activity plot. $c_{\mathrm{Tolaasin\ II}}$

(µM) is plotted as a function of lipid concentration, c_{lipid} (µM). Colored lines represent fitted equi-activity lines for 20% - 80% leakage (see color code in figure).

Table 2.9: Data of equi-activity plot for POPC LUVs. The total tolaasin II concentrations, $c_{\text{Tolaasin II}}$, needed to cause a certain degree of calcein leakage (L=X%) at each lipid concentration. Concentrations are given in µM.

c_{POPC} (µM)	$c_{\text{Tolaasin II}}$ (µM)						
	L=20%	L=30%	L=40%	L=50%	L=60%	L=70%	L=80%
30	0.055	0.072	0.095	0.124	0.162	0.215	0.291
60	0.119	0.142	0.164	0.192	0.227	0.276	0.339
100	0.192	0.227	0.273	0.32	0.365	0.426	0.495
200	0.332	0.394	0.465	0.544	0.633	0.725	0.841
300	0.587	0.683	0.775	0.891	1.017	1.165	1.326

A linear regression of each equi-activity line, shown in Figure 2.5, panel B, was performed using the software OriginPro 2019. Table 2.10 presents the fitting data for the y-intercepts ($c_{\text{Tolaasin II}}^{aq}$) and the slope ($R_e$) with respective SD. Grey shadings highlight the fit data with the lowest correlation between individual data points from the leakage data set. Only these were considered in the analysis of the partitioning coefficient. Free fitting of data points at L=20% yielded in impossible negative $c_{\text{Tolaasin II}}^{aq}$. This is due to the very small concentration range which makes the numbers indistinguishable from zero. To still obtain values for R_e, data of equi-activity line at L=20% were fit with the y-intercept set to zero.

Table 2.10: Results of equi-activity analysis with POPC LUVs. $c_{\text{Tolaasin II}}^{aq}$ and R_e with SD derived by linear regression of the equi-activity lines with POPC LUVs. Depicted numbers are rounded to four decimal places.

Leakage (%)	$c_{\text{Tolaasin II}}^{aq}$ (µM)	SD of $c_{\text{Tolaasin II}}^{aq}$ (µM)	R_e	SD of R_e	R-Square
20	0	-	0.0019	0.0001	0.9938
30	0.0023	0.0235	0.0022	0.0001	0.9842
40	0.0157	0.0218	0.0025	0.0001	0.9892
50	0.0290	0.0233	0.0028	0.0001	0.9905
60	0.0470	0.0245	0.0031	0.0001	0.9917
70	0.0777	0.0323	0.0035	0.0002	0.9883
80	0.1256	0.0397	0.0039	0.0002	0.9855

3 Lifetime plot

If tolaasin II were to act according to an all-or-none leakage mechanism, individual calcein-LUVs would remain completely intact, while others would lose their complete content. As the lifetime of the fluorophore calcein correlates with its local concentration according to the Stern-Volmer relationship, $\tau = \tau_0/(1 + K_D \cdot c_E)$, the lifetime of the entrapped calcein population would always remain constant, completely independent of how much leakage had already occurred throughout the sample.

A lifetime plot describes the correlation between peptide-triggered vesicle leakage and the lifetime of the entrapped fluorophore on a reciprocal scale. Here, we encapsulated 70 mM calcein, c_E = 70 mM, into the tested vesicles, yielding with a dynamic quenching constant, K_D, of 0.13 mM (Bassarello et al., 2004; Patel et al., 2009) a corresponding lifetime of 0.4 ns. For all-or-none leakage, all data points with increasing leakage must therefore be located on a vertical line at 0.4 ns (Ladokhin et al., 1995; Patel et al., 2009).

Figure 3.1 shows calcein leakage after 1-hour incubation as a function of the lifetime of the entrapped calcein on a reciprocal scale. Data points describe with increasing calcein leakage a rather slightly upward bent curve and clearly no vertical line at 0.4 ns. Hence, we may conclude that tolaasin II does not induce membrane permeabilization by an all-or-none mechanism in any of our tested model membranes. This finding is also independent of membrane thickness.

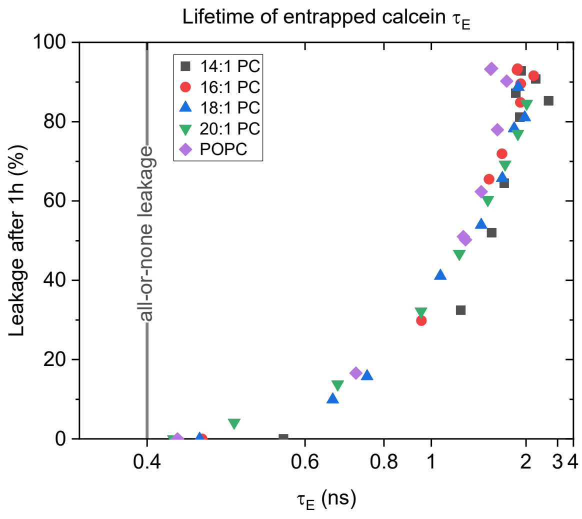

Figure 3.1: Calcein leakage as a function of the lifetime of the entrapped calcein population, τ_E, on a reciprocal scale, of the tested calcein-LUVs (see color code in figure). Data points were derived after 1-hour LUV incubation with tolaasin II.

4 Schematic representation of tolaasin-induced monolayer curvature stress

Monolayer curvature stress results from molecules with a polar part that requires more (positive curvature) or less (negative) interfacial area than their hydrophobic part. The intrinsic area of the hydrophobic part in a given membrane is the ratio between its hydrophobic volume and the hydrophobic thickness of a membrane. The cartoon illustrates that the space to be filled by the hydrophobic part of the peptide (light yellow range) is larger than its true size. This becomes even more pronounced for a thicker membrane.

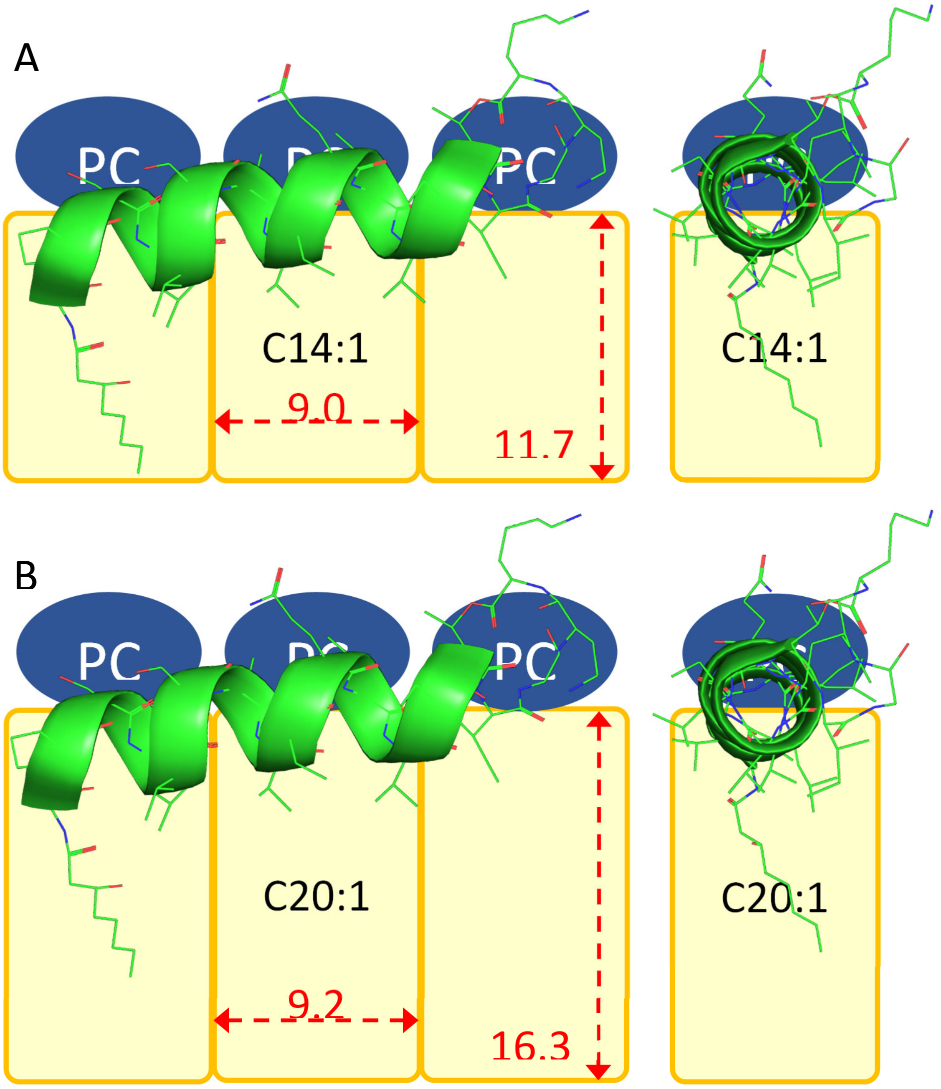

Figure 4.1: Schematic representation of tolaasin incorporation into the membrane. Panel A shows the dimensions of tolaasin in a ~~C~~14:1 PC membrane and the resulting packing differences after incorporation of the peptide. Panel B shows the incorporation of tolaasin into the ~~C~~20:1 PC bilayer, respectively. Numbers for lipid area calculation were taken from Marsh, 2013 and Nagle and Tristram-Nagle, 2000, the effective diameters of lipids shown in the cartoon correspond to cylinders of the hydrophobic volume at a given thickness.

5 References

Bassarello, C., Lazzaroni, S., Bifulco, G., Lo Cantore, P., Iacobellis, N. S., Riccio, R., et al. (2004). Tolaasins A−E, Five New Lipodepsipeptides Produced by Pseudomonas tolaasii. *J. Nat. Prod.* 67, 811–816. doi: 10.1021/np0303557.

Ladokhin, A. S., Wimley, W. C., and White, S. H. (1995). Leakage of membrane vesicle contents: determination of mechanism using fluorescence requenching. *Biophys J* 69, 1964–1971. doi: 10.1016/S0006-3495(95)80066-4.

Marsh, D. (2013). *Handbook of lipid bilayers*. 2nd ed. p. 381: CRC Press, Taylor & Francis Group, LLC.

Nagle, J. F., and Tristram-Nagle, S. (2000). Structure of lipid bilayers. *Biochim Biophys Acta* 1469, 159–195.

Patel, H., Tscheka, C., and Heerklotz, H. (2009). Characterizing vesicle leakage by fluorescence lifetime measurements. *Soft Matter* 5, 2849–2851. doi: 10.1039/b908524f.
